# The Social Dimension of the European Union: A Means to lock out Social Competition?

**DOI:** 10.1007/s11205-022-03012-6

**Published:** 2022-12-02

**Authors:** Javier Bilbao-Ubillos

**Affiliations:** Department of Applied economics, University of the Basque Country (UPV/EHU), Avda. Lehendakari Agirre, 83, 48105 Bilbao, Spain

**Keywords:** European Union, Social dimension, Fiscal sustainability, Social competition, Inequalities in social protection levels, Conditionality of EU funding

## Abstract

In the process of European integration, the social dimension has to date been subordinated to presumed economic requirements. There have been no specific commitments to preserve the European Social Model (ESM), which has been gradually diluted as a result of successive EU enlargements, the impact of ageing, job insecurity and socio-cultural changes on social protection systems and the pressure exerted by globalisation in a context of ever harsher market competition. Moreover, the decisions adopted by the EU in addressing the 2008 economic crisis have led Community institutions gradually to impose reforms of pension systems and labour markets, particularly in southern countries, which threaten to reduce the level of protection provided for citizens. This paper argues that the EU aquis is not sufficient to prevent social competition and warns of the risks arising from the current dynamic in terms of maintaining social cohesion, equal opportunities and fairness as basic features of European identity. Some data that illustrate the varying degrees of intensity and effort in social welfare provisions by Member States and the gradual divergence in social protection benefits are provided (using dispersion measurements). In an effort to be proactive, a number of proposals are given with a view to reversing this trend and consolidating the social content of the European project.

## Introduction

Over and above speeches and rhetoric (“Improving working conditions, living standards and gender equality have been central objectives of the EU” (European Commission, 2017, p. 6)), the common institutions of the European Union (EU) have never held significant competences in core social protection matters. Under the principle of subsidiarity, such matters have remained in the hands of its Member States (MS). The setting of benchmark protection standards and the adopting of harmonisation measures have not been envisaged in the gradual design of European integration, so social protection systems have been subject to market discipline: those MS with more public resources and higher levels of relative competitiveness have been able to fund more provisions, and the rest have done what they could.

In words of Jones et al., (2016, p. 1010), “The single currency centralised monetary authority but provided only weak coordination of fiscal policy and no obvious mechanism to facilitate macroeconomic adjustment within the Member States”. The incompleteness of European economic and monetary integration led inexorably to a crisis in the Euro, which was exacerbated by an inadequate response on the part of Community institutions (Bilbao-Ubillos, 2013).

In matters of social protection politics really matters (Alsasua et al., 2007), and each MS has set its political priorities in line with its own political and cultural traditions and specific preferences concerning the bond of solidarity and cohesion. But with the onset of the crisis and the associated sovereign debt problems any room for manoeuvre in decision-making has decreased: on the one hand countries face different levels of borrowing (and financial market scrutiny) and on the other hand Community institutions have continued to ramp up the pressure in regard to their agendas to *modernise* social protection.

Indeed, in the past twenty years the EU’s recommendations have all been for enhanced efficiency and financial sustainability in national social protection systems and more flexible labour markets in MS as a way of encouraging competitiveness and consolidating public finance in the long term. Public pension systems have been a priority target in the recommendations for reforms made by EU institutions to MS. According to the EU White Paper on Pensions: “It has thus become more urgent than ever to develop and put in place comprehensive strategies to adapt pension systems to changing economic and demographic circumstances” (European Commission, 2012, p. 1). This has been implemented through reforms that have sought to bring the parameters of the systems into line with fiscal sustainability, or through structural reforms such as shifting from defined benefit to defined contribution schemes or establishing mandatory funded pillars. As Blank highlights, “replacement rates have been lowered in such a way that the former core beneficiaries can no longer rely solely on the social insurance systems if they want to maintain their standard of living” (Blank, 2020, p. 513).

In spite of this, the Commission’s discourse takes on board “the need to strengthen the link between economic, social and environmental development, on the fact that inequalities hold back economic development, and on the need to build a more inclusive growth model […] to ensure a level playing field, limit the risk of social dumping or “race to the bottom”, and facilitate economic and social integration” (European Commission, 2016, p.3–4). In other words the Community’s rhetoric is clearly inconsistent with its tangible initiatives on social issues.

This paper is organised as follows. Section Two looks at the space allocated to EU social policy in the process of European integration and examines the concept of social competition. Section Three ties the various enlargements of the EU and the associated new social demands to the growing heterogeneity of social protection systems of MS. Section Four considers the effects of the 2008 crisis in terms of the conditions imposed for the provision of EU aid and the knock-on adjustments in the social policies of MS in the context of austerity policies. That conditionality has been reinforced by Community actions in response to the recession arising from COVID-19. Finally, Section Five sums up the main conclusions of the paper and gives a number of proposals for preventing the social content of the European project from being watered down.

## The Space for EU Social Policy and the Concept of Social Competition

As summed up by Šmejkal (2015), Title X of the Treaty on the Functioning of the EU, entitled “Social Policy”, that could have contained a specific mandate and set of directly claimable social rights, was not changed by the Lisbon Treaty (which came into force in 2009), and thus does not allow the EU to do more than “support and complement” the activities of the MS in the fields of labour and social security law. In all key issues (social security and the social protection of the workers) the MS have retained the right of veto in the Council.

The main instruments of the EU for establishing social rights and social protection benchmarks, such as the European Pillar of Social Rights (European Parliament, the Council of the European Union and the European Commission, 2017), are not binding but operate merely as guidelines and recommendations. In fact, the only significant EU actions have been certain qualitative interventions in matters such as the free circulation of workers, the transferability of pension rights, equality in the treatment of men and women in the workplace, health, safety and hygiene in the workplace, the right of information and consultation of workers, minimum working hours (undemanding) and social dialogue. The directives in place in these areas are binding on MS, and they all seek to facilitate the movement of production factors throughout the EU. In other words they reinforce the idea that social issues are subordinate to economic issues in the EU, an idea that can already be seen in certain official reports (Department for Business, Innovation and Skills of the British Government, 2013, p. 10).

The only social issue on which action has been taken more recently by Community institutions is the fight against poverty and social exclusion, in the framework of the European Semester and the 2020 European Strategy.

As Barbier (2012, p. 381) states, social policy and economic policy are seen as separate fields and the former is generally accepted to be legally and explicitly subordinate to the latter. Streeck (1995) develops this argument by stating that the economic and market nature of European integration, as set out in the Treaties, favours a form of social policy that is oriented to integrating the EU labour market and mandates the use of social policy mainly to enable efficient market functioning.

But the path followed by European integration has not been linear, or indeed entirely planned; rather, there have been jumps and gaps, usually as the European project faced new challenges. The 2008 crisis was the most critical challenge of all. In the words of Jones et al. (2015, p. 1012), “at moments when the crisis intensified and the monetary union appeared vulnerable, EU leaders opted to do what they thought necessary to save the Euro, but nothing more. Taken together, the series of incremental reforms adopted sequentially in response to the crisis— steps including establishing bailout funds, tightening fiscal surveillance and moving toward banking union— has led to one of the most rapid periods of deepening of integration in EU history”.

The intergovernmentalism of the process of European integration (Moravcsik, 1998) explains why, in moments of crisis, negotiations between EU leaders produce lowest common denominator bargains that yield only incremental reforms rather than comprehensive ones. And since the efforts to tackle the crisis from 2010 onwards those reforms have affected the subordination of social policy to economic policy.

The consensus in the literature before the crisis was that the EU’s ability to impose specific welfare state reforms was very limited (Hassenteufel & Palier, 2015), but in its aftermath Community institutions have found themselves holding new tools for persuasion to get their recommendations heeded, as detailed below. As Copeland and Daly (2018) conclude, after analysing the country-specific recommendations made by EU institutions for 2011–2015 from a market-correcting versus market-making perspective, there is a clear lack of agreement among Member States and their representatives over the most appropriate direction for EU social policy and lack of commitment towards a role for EU social policy in correcting for market outcomes.

Vaughan-Whitehead (2017) also argues that fiscal consolidation policies have accelerated such changes, and led to questions about the sustainability of a number of elements of the European Social Model.

The present paper starts from the idea of social competition as per Maslauskaitė (2013), in the sense that cost competition among Member States might put pressure on social systems, leading to a “race to the bottom”. It is often argued that the tensions between free market competition and the ESM are eminent in the environment of significant heterogeneity among different MS. And this idea of social competition is built on the prior notion of social dumping, which “usually refers to alleged unfair or uncompetitive advantage gained due to differences in social protection, social regulations and social conditions between sectors and countries” (European Centre for International Political Economy, 2017).

However, this paper also places the concept of social competition in the context of the constraints arising from the need for public funding on the part of MS: their dependence on Community funds and the ECB’s sovereign bond purchases leaves dependent countries in a position of weakness in the face of recommendations by the Commission or the Council, which frequently call for reforms of public pension systems and labour markets in MS.

Weakness in competitive terms and budget imbalances in MS increase their vulnerability, which in turn fosters social competition, in the first case as a national strategy and in the second as a measure induced by Community institutions in exchange for European aid.

## EU Enlargements, Further Social Demands and Increasing Heterogeneity of Social Protection Systems

As more members joined the EU, the economies of Member States became increasingly heterogeneous and greater differences emerged between social protection measures for EU citizens in different countries. In spite of the rhetoric concerning the European Social Model (ESM), it comes as no surprise that in 2019 citizens of Luxembourg received an average of €22.172 per head in social protection services (at current prices), while for Bulgarians the figure was just €1.460, i.e. less than 1/15 as much (Eurostat, 2022a). It is clear that EU citizenship alone does not provide equivalent protection in all Member States. Table 1 shows the trend in per capita spending on social protection in the EU since 1991 in real terms, highlighting the magnitude and persistence of the differences.

**Table 1:** Total expenditure on social protection per head of population in the EU (Euro per inhabitant at constant 2010 prices), 1991–2019

COUNTRY	1991	1995	2000	2005		2010	2015	2019	
**EU-28**	n.a.	n.a.	n.a.	n.a.		7.289,81	7.567,63	7.940,08	
**Belgium**	n.a.	n.a.	7.855,78	8.921,42		9.849,13	10.180,33	10.301,33	
**Bulgaria**	n.a.	n.a.	n.a.	571,02		879,26	1.052,14	1.183,02	
**Czechia**	n.a.	1.653,08	2.063,56	2.553,71		2.998,05	3.079,80	3.456,81	
**Denmark**	9.599,84	11.315,8	11.524,93	12.902,19		14.921,55	15.508,64	15.672,94	
**Germany**	6.505,26	7.534,18	8.446,83	8.611,63		9.417,39	10.178,89	11.133,36	
**Estonia**	n.a.	n.a.	963,21	1.322,01		1.944,88	2.199,20	2.629,76	
**Ireland**	n.a.	4.030,30	5.165,07	6.969,58		9.228,80	8.517,11	8.970,55	
**Greece**	n.a.	2.807,40	3.099,50	4.179,55		5.264,56	4.401,98	4.423,30	
**Spain**	n.a.	3.654,48	4.085,62	4.798,64		5.715,80	5.434,71	5.785,43	
**France**	6.880,21	7.685,37	8.300,61	9.226,33		10.215,20	10.897,83	11.296,70	
**Croatia**	n.a.	n.a.	n.a.	n.a.		2.233,83	2.278,63	2.711,62	
**Italy**	n.a.	5.785,09	6.472,42	7.112,01		7.809,69	7.639,64	8.056,68	
**Cyprus**	n.a.	n.a.	2.700,43	3.865,01		4.368,05	4.187,27	4.595,42	
**Latvia**	n.a.	n.a.	798,03	985,55		1.549,36	1.646,64	1.999,33	
**Lithuania**	n.a.	n.a.	835,16	1.107,84		1.730,54	1.858,02	2.360,89	
**Luxembourg**	n.a.	10.584,8	12.518,83	15.735,33		17.870,51	18.530,16	18.783,31	
**Hungary**	n.a.	n.a.	1.562,95	2.151,60		2.225,83	2.163,71	2.267,76	
**Malta**	n.a.	n.a.	2.239,04	2.552,96		3.094,41	3.366,69	3.548,13	
**Netherlands**	n.a.	8.388,10	8.539,62	9.381,45		11.285,56	11.522,03	11.889,27	
**Austria**	n.a.	8.139,72	8.925,99	9.454,52		10.478,93	10.674,06	10.958,73	
**Poland**	n.a.	n.a.	1.264,03	1.498,87		1.874,38	2.130,64	2.797,71	
**Portugal**	n.a.	2.719,15	3.347,35	3.918,79		4.386,00	4.304,21	4.570,69	
**Romania**	n.a.	n.a.	369,78	606,10		1.083,57	1.090,07	1.477,77	
**Slovenia**	n.a.	n.a.	3.398,77	3.842,20		4.322,33	4.319,15	4.687,65	
**Slovakia**	n.a.	1.345,12	1.647,95	1.732,58		2.285,56	2.436,30	2.665,95	
**Finland**	7.384,46	8.055,42	7.926,60	9.019,96		10.223,45	10.990,82	11.330,37	
**Sweden**	n.a.	9.569,54	9.735,21	10.944,24		11.322,94	12.247,47	12.064,41	
**United Kingdom**	n.a.	5.352,92	6.215,20	7.923,72		8.496,06	8.713,43	8.505,64*	

n.a.: Not available; * Data for 2018

Source: Eurostat (2022b). Data extracted on 07/07/2022

These differences in social protection levels do not stem only from differences in income levels between countries but also from political preferences, as reflected in the effort put into social protection: for instance, as shown in Table 2, in 2019 Denmark earmarks 31.5% of its annual GDP for social protection spending, while Ireland earmarks just 13.6% (Eurostat, 2022b).

**Table 2:** Total expenditure on social protection as % of GDP in the EU, 1995–2019

Country	1995	1999	2003	2008	2013	2019	
European Union*	26.4	25.7	26.7	26.0	27.8	28.0	
**Belgium**	27.0	26.0	27.1	27.9	28.8	28.8	
**Bulgaria**	n.a	n.a	n.a	14.7	17.0	16.5	
**Czechia**	16.0	17.8	18.5	17.8	19.6	18.8	
**Denmark**	31.4	29.2	30.1	30.4	33.0	31.5	
**Germany**	27.6	28.7	29.9	27.3	28.0	30.1	
**Estonia**	n.a	15.3	12.5	14.6	14.6	16.6	
**Ireland**	18.2	14.2	16.7	20.9	22.0	13.6	
**Greece**	19.1	21.4	18.6	22.8	25.8	25.1	
**Spain**	21.0	19.3	19.8	21.6	25.5	24.1	
**France**	30.0	29.5	30.5	30.8	31.9	33.5	
**Croatia**	n.a	n.a	n.a	18.7	21.0	21.3	
**Italy**	23.1	23.7	24.5	26.3	28.5	29.2	
**Cyprus**	n.a	n.a	16.7	17.6	20.9	18.1	
**Latvia**	n.a	16.7	13.3	12.0	14.4	15.6	
**Lithuania**	n.a	16.3	13.4	15.9	14.5	16.5	
**Luxembourg**	20.5	19.8	21.8	19.9	22.7	21.9	
**Hungary**	n.a	20.3	20.9	22.3	20.5	16.7	
**Malta**	15.8	17.2	17.2	18.0	18.8	14.7	
**Netherlands**	28.4	24.8	26.0	26.1	28.8	28.8	
**Austria**	28.9	28.4	28.7	27.6	28.8	29.3	
**Poland**	n.a	n.a	20.9	19.4	19.1	21.3	
**Portugal**	20.1	20.4	22.8	23.4	26.0	24.0	
**Romania**	n.a	n.a	13.4	13.7	14.6	15.3	
**Slovenia**	n.a	23.5	23.3	21.0	24.2	22.2	
**Slovakia**	18.1	19.8	18.0	15.7	17.8	17.9	
**Finland**	30.6	25.4	25.5	25.0	30.2	30.1	
**Sweden**	32.0	28.9	30.1	27.7	29.4	27.6	
**United Kingdom**	n.a	n.a	25.1	25.5	27.7	25.7¹	

* EU-15 from 1995 to 2003; EU-27 from 2008 to 2019; ¹ Data for 2018

n.a.: Not available

Source: Eurostat (2022b). Data extracted on 07/07/2022

The European Commission itself, in its *Reflection Paper on the Social Dimension of Europe*, states that “social realities within Europe differ greatly, depending on where we live and work” (European Commission, 2017, p. 8). The same document also states that “beyond the labour market, EU-27 countries also display a variety of welfare and social protection systems in terms of political preferences and budgets” (European Commission, 2017, p.11).

But diversity is not the only problem of the ESM: globalisation (with more and more raw exposure to market dynamics and new, emerging actors), new socio-cultural values, new ways of living with greater diversity, the ageing population, pandemics, increasing inequality, tax avoidance by large taxpayers, lower quality jobs (effects of insecure jobs and in-work poverty) and economic stagnation are all problems that must be tackled by European welfare states (Bilbao-Ubillos, 2021). Those states are tempted to reduce their domestic protection levels in order to make themselves artificially more competitive relative to other MS and rivals. There are no Community mechanisms to prevent or hinder the adoption of such downgrading measures (indeed, they are rather encouraged), though with the outbreak of the current pandemic Community bodies have expressed concern at the effects of the crisis on fairness and on poverty, and in terms of the resilience of health systems.

## Effects of the 2008 Crisis: Conditionality and Adjustments in Social Policy in Times of Austerity

The economic crisis that hit the EU in 2008, with the subsequent double-dip in 2011 (due largely to poor crisis management by Community institutions) not only led to a substantial contraction of the economy and employment in EU economies:

- According to Eurostat (2021), at the end of 2014 the GDP of the Eurozone was still below its 2008 level in real terms. The MS with the biggest cumulative drops in GDP between 2008 and 2014 were Greece (28.3 points), Croatia (13), Cyprus (13), Spain (7.-9), Italy (7.8), Portugal (7.5), Slovenia (7.2) and Latvia (7).
- For 111 of the 280 EU regions, GDP per inhabitant remained below its 2008 level for between three and eight years (Eurostat, 2019).

There were also increases in inequality and poverty within countries (De Beer, 2012), and the crisis had a highly substantial impact on the quality-of-life of citizens: a study estimates that standardised quality-of-life decreased sharply in 18 of the 27 MS, with the sharpest drops in Latvia and Greece, followed by Lithuania, Spain, Romania, Estonia, Poland and Slovakia (Somarriba et al., 2015). The same study indicates that the crisis hit the EU countries of eastern and southern Europe harder than those of the centre and north.

The austerity-based EU crisis exit strategy consolidated in 2010 was pro-cyclical, which resulted in a double-dip in 2011 and encouraged cutbacks in the social provisions of MS. As a percentage of GDP, the cuts in social spending over the period 2010–14 were largest in Bulgaria, Greece, Hungary, Ireland, Romania, Slovakia and the Baltic countries – all of which were already underperforming on labour market and social protection indicators (Leschke et al., 2012).

But the 2008 crisis did not only give rise to inequality, unemployment, new social needs (which exerted pressure on national social protection systems) and one-off cutbacks in social spending, but also to a substantial change in the extent to which Community institutions were able to impose a political agenda on those countries whose economies performed worst in the crisis. This showed up in growing problems of public debt and rising interest premiums. As the situation grew worse in 2010 and 2011 and countries began to call for aid from European and international institutions, the EU sought to set up instruments and institutions to improve the financial situation of countries with problems, and in return required that they carry out a number of reforms which would theoretically enable them to stabilise their economies, at least in the long term (Leschke & Jepsen, 2012).

On the one hand, as noted by La Porte & Heins (2015), the new instruments created between 2010 and 2013 to reinforce the governance of the Economic and Monetary Union (the European Semester on coordination of economic policies, with further content added and a toughening up of the Six-Pack, Fiscal Compact and Two-Pack measures, updating the Stability and Growth Pact or the Europe 2020 strategy) strengthened budgetary discipline and established exhaustive supervision of national policies. This stricter supervision even meant that the national budget plans of Eurozone countries now have to be submitted to the Commission and the Eurogroup for approval before they are formally enacted in the legislative chambers of each MS. Based on the analysis conducted by the Commission, the Council issues a ruling before each MS can establish its final budget for the following year.

Stability programmes (for Eurozone countries) and convergence programmes (for non-Eurozone MS) setting out medium-term budget targets must be submitted to the European Commission. These programmes are assessed by the Commission and are subject to Country Specific Recommendations from the Council, which mainly involve major structural reforms and, in particular, pension system reforms and increased labour market flexibility. In general, as stated by Crespy & Menz (2015), social policy concerns have been further marginalised in these new governance arrangements.

On the other hand, the Community financial aid received (first through the EFSM and later the ESM) by Eurozone countries with severe sovereign debt problems was made conditional on their governments implementing a tough programme of economic adjustments negotiated with the Commission the ECB and the IMF. Thus, countries bailed out with Community funds were obliged to sign a memorandum of understanding which included, among other things, clear commitments to reform labour legislation, pension systems, unemployment provisions and their public sectors (including privatisations and wage cuts for public employees). Greece, Ireland, Portugal and Cyprus were bailed out (and Spain was partially bailed out), while Hungary, Latvia and Romania (all three of which were non-Eurozone countries at the time, though Latvia joined the Euro on 1 January 2014) requested financial aid from international institutions. Greece’s three bailouts were linked to the toughest conditions, though those applied in the case of Portugal were also substantial (Theodoropoulou, 2015). In the words of López Escudero (2015), the degree of intrusion into state authority on economic and budgetary policy was enormous.

Those countries which were not bailed out but which nevertheless needed to use the ECB’s sovereign debt purchase programmes were also obliged to meet certain implicit conditions, in a system known as soft conditionality (Sacchia, 2015). This system was used in Italy, Spain and elsewhere, and was based on an implicit understanding of the challenges or sanctions that might ensue if the suggestions of the Community were not followed, in a context of asymmetry of power and a pressing need on the part of these countries for funding (at a time when they had trouble placing their sovereign debt bonds on the markets). Thus, the (strictly confidential) letters sent by Trichet and Draghi to the prime ministers of Italy and Spain on 5 May 2011 included specific requests for adjustments and reforms (“a need for further significant measures to improve the functioning of the labour market” in the case of Spain; and “a thorough review of the rules regulating the hiring and dismissal of employees” and “to intervene further in the pension system, making more stringent the eligibility criteria for seniority pensions and rapidly aligning the retirement age of women in the private sector to that established for public employees” in that of Italy). These letters were answered the following day^1^ with specific commitments so that the ECB would purchase sovereign bonds and bring down the risk premium, which had rocketed.

As indicated by Hassenteufel & Palier (2015), with the deterioration of the financial situation in France (a deficit of 7.5% of GDP by 2010) the balance of forces in the relationship between the French government and EU institutions also changed. At the end of May 2013, the Commission granted France two additional years to correct its excessive deficit. In the Recommendation that followed this negotiation, published on 18 June 2013, the Council intended that the budgetary measures the French government announced must be effectively implemented. The Council concluded explicitly that the French authorities should strengthen the long-term sustainability of the pension system by further adjusting all relevant parameters (and in January 2014 France adopted a new pension reform law). The Council’s recommendation also concerned labour market reforms: lowering labour costs, better integration of the youngest and oldest workers, reducing the segmentation of the labour market and more flexibility in the firing and hiring regulation.

The conclusion that can be reached is in line with the words of Barbier (2012, p. 391): “Largely irrespective of partisan orientation, governments have systematically responded to the crisis and its consequences on public finances, deficits and debts by imposing social protection cuts and containment measures”. It should therefore be no surprise that, as shown in Table 1, numerous MS on the periphery of Europe (e.g. Greece, Ireland, Spain, Italy, Cyprus, Hungary, Portugal and Slovenia) reduced their per capita social protection provisions (in real terms) in 2015 to below 2010 levels, in spite of increases in unemployment, early retirements and social need as a result of the crisis. The four countries bailed out, plus three more with unique public funding needs, resorted to cutting back social provisions, urged on by Community institutions. Following this trend towards the use of fiscal sustainability criteria in welfare states, core EU countries such as Belgium, Denmark and Sweden began to reduce the annual funding provisions for social protection for their citizens from 2018 onwards.

And just when the economies of the EU had recovered their pre-crisis levels, the social and health consequences of the COVID-19 pandemic (with large numbers of people dead and ill) and its economic consequences placed society and national social protection systems under strain once more. The 2020 annual report of the EU’s Social Protection Committee states that income inequalities are expected to increase in at least nine MS in spite of the measures taken as a political response to the crisis. The countries where the increase in inequality (measured according to the Gini Index) is likely to be greatest include Malta, Estonia, Spain, Slovakia and the Netherlands (Social Protection Committee, 2020, p.41). The poverty rate measured according to the AROPE index is set to rise in all the countries analysed in the report, with the biggest increases expected in Spain (uniquely affected because of the weight of tourism in its economy), Hungary, Malta, Estonia and Slovakia (Social Protection Committee, 2020, p.42).

In the case of Spain Aspachs et al., (2020) use big data techniques to analyse over 3 million monthly wage payments, and confirm that the crisis is hitting Spanish society extremely hard but unevenly. For February and April the observe, even before taking public sector transfers into account, that the percentage of individuals with no income increased by 15 points and one third of individuals suffered a decrease in income (with 13% dropping into the lower income bracket and 20% losing their income entirely). Finally around 30% of those in the higher income bracket also suffered a decrease in income, with 20% dropping into the middle income bracket and a small number into the low income and no income brackets.

In an attempt to counteract the economic and social impacts of this health crisis, Community institutions have created financial instruments to support SM such as the Coronavirus Response Investment Initiatives (CRII I & II) and Temporary Support to mitigate Unemployment Risks in an Emergency (SURE). They have also activated the general escape clause in the Stability and Growth Pact to give MS exceptional budget flexibility so as to mitigate the social economic consequences of the pandemic. However, six months after its creation the EU’s most powerful tool for tackling the crisis has not yet been deployed: the Anti-Covid Recovery Fund (a Next Generation EU recovery fund with a provision of €750 billion to be distributed based on objective need and not on the economic or demographic weight of each country). This fund was finally released by the European Council on 10 December 2020, and its effects will presumably begin to be felt in the spring of 2021.

But this instrument reinforces the new tendency to introduce strong conditions in EU programmes: in order to receive this financial aid, MS must prepare national recovery and resilience plans setting out their programmes of investment and reforms for 2021-23. These detailed plans must be sent to Brussels for them to be eligible for aid, along with proposals for reforms based on the recommendations of the EU authorities. In the case of Spain these include reforms of pensions, taxation, the labour market and education and even changes in the social protection model to tackle the levels of chronic poverty carried over in the country from the previous crisis.

Structural reforms of pension, unemployment protection and labour market systems have become a regular EU recommendation and a prerequisite for eligibility for Community funds. They can therefore be seen as new tools for persuasion in the hands of Community institutions.

Paradoxically, however, the EU urges MS to reform essential elements of their welfare states with the excuse of ensuring their financial sustainability, but allows some of its members (specifically Ireland, the Netherlands, Malta, Cyprus, Belgium and Luxembourg) to set up advantageous tax systems for large taxpayers (low effective tax rates, possibilities for tax deductions) and to design ad hoc tax arrangements for multinationals in order to attract investments, thus depriving countries with public account imbalances of public resources. It must also be pointed out that fiscal competition between MS and this flight of capital are made possible by the lack of any harmonisation of tax systems in the EU.

Tørsløv et al., (2018) estimate that $616 billion in profits made by foreign companies in 2017 (35% of the total amount) was shifted to other tax jurisdictions outside the company’s home country. Ireland (106 billion), the Netherlands (57), Luxembourg (47), Belgium (13), and Malta (12) top the list of those jurisdictions. Tax competition redistributes tax bases across countries: According to their estimates, under a perfect tax harmonization scenario, profits would be 17% higher than they currently are in France and 14% higher in the UK (Tørsløv et al., 2020). The Commission estimates the total loss of revenue (including aggressive fiscal planning by multinationals) to be in excess of €1 trillion per annum.

This is a form of unfair tax competition which is not combated as firmly as other forms of unfair competition within the EU. It is draining off public resources crucial to other countries, which are often therefore forced to cut back on provisions to their citizens.

Nor should it be any surprise that the weak process of convergence in spending on social protection provisions that began during the economic upturn phase prior to 2008 ground sharply to a halt as a result of the crisis and the way in which it was handled by Community and national institutions. In terms of sigma convergence, Tables 3 and 4 show that the standard deviation for spending on social protection in the EU gradually increased, reaching its peak in 2019:

**Table 3:** Main statistics for the expenditure on social protection per head of population (Euro per inhabitant at constant 2010 prices), 1991–2018

	1991	1995	2000	2005	2010	2015	2019
**Number of observations**	4	17	26	27	28	28	28
**Mean**	7.592,44	6.246,34	5.000,10	5.625,51	6.324,13	6.483,91	6.790,16
**Median**	7.132,34	6.659,64	3.742,20	4.179,55	4.825,28	4.360,57	4.641,54
**Standard deviation**	174,27	325,86	360,56	389,73	420,80	426,09	434,73
**Max. value** **(country)**	9.599,84 (Denmark)	11.315,8 (Denmark)	12.518,83 (Luxemb.)	15.735,33 (Luxemb.)	17.870,51 (Luxemb.)	18.530,16 (Luxemb.)	18.783,31 (Luxemb.)
**Min. value** **(country)**	6.505,26 (Germany)	2.719,15 (Portugal)	369,78 (Romania)	571,02 (Bulgaria)	879,26 (Bulgaria)	1.052,14 (Bulgaria)	1.183,02 (Bulgaria)

Source: Own elaboration based on Eurostat (2022b). Data extracted on 07/07/2022

**Table 4:** Main statistics for the expenditure on social protection as % of GDP, 1995–2019

		1995		1999		2003		2008		2013		2019
**Number of observations**		17		22		26		28		28		28
**Mean**		23,99		22,12		21,74		21,59		24,09		22,67
**Median**		23,10		20,90		21,35		21,30		23,80		22,05
**Standard deviation**		5,73		4,94		5,73		5,31		6,03		6,09
**Max. Value (country)**		32.0 (Sweden)		29.5 (France)		30.5 (France)		30.8 (France)		33.0 (Denmark)		31.5 (Denmark)
**Min. Value (country)**		15.8 (Malta)		14.2 (Ireland)		12.5 (Estonia)		12.0 (Lavtia)		14.4 (Lavtia)		13.6 (Ireland)

Source: Own elaboration based on Eurostat (2022b). Data extracted on 07/07/2022

- Looking at per capita spending on social protection (Table 3), the standard deviation is shown to have risen from 174 to 1991 to 434 in 2019, i.e. to have more than doubled in 28 years. The standard deviation is very high because this is an asymmetrical distribution in which the mean (which is highly sensitive to increasingly polarized observations) and the median (which is more robust) move increasingly apart. Furthermore the size of the variable observed is very large. The minimum and maximum figures for each year have been added to the table to show the increasing polarization of the data observed. This is what underlies the sustained increase in the standard deviation, showing the strongly divergent trend in per capita spending on social protection in the EU;

- Looking at spending on social protection as a percentage of GDP (Table 4), the standard deviation has increased from 5.73 (1995) to 6.06 (2019). In this case the standard deviations are lower because the size of the variable observed is also smaller, but again the greatest dispersion in recent years is found in 2019.

This divergent trend in terms of both social protection intensity and effort may be due in part to the successive enlargements of the EU, but a significant part of it can also be attributed to the uneven trend in national networks of social protection available to EU citizens (data for almost all the current MS have been incorporated into the statistics since 2000, but the standard deviation continues to increase).

## Conclusions and Proposals

After decades of inaction in regard to social protection given the type of architecture of European integration designed, the EU is now adopting guidelines in its economic and social policy recommendations that threaten the level and coverage of national social protection systems and working conditions, particularly in those countries which are most financially dependent (i.e. those in most need of Community funds and ECB programmes). This trend is not subject to specific scrutiny by European citizens and could drain the social dimension of the EU of all tangible content, thus preventing minimal cohesion among citizens.

This paper argues that the European aquis is not sufficient to prevent social competition and concludes that the way in which the 2008 crisis was handled has reinforced the subordination of social policy to economic policy. To shore up the social dimension of the European project, I would like to make the following proposals:

1. Set up a specific discussion on minimum standards of social protection and labour rights considered essential for European citizenship. The limited tools for legislative intervention available to the EU on social issues include the setting of minimum requirements and the harmonisation of basic standards (European Commission, 2017). Community institutions need to prevent any initiatives by MS to go below those minimum standards of protection.
2. Over and above setting such minimums, if it is considered essential to reform pension and unemployment protection systems in certain MS then Community funds should be provided to make up shortfalls in national provisions and ensure sufficient income for retired and unemployed individuals in countries with less generous protection systems in the wake of imposed or recommended reforms. Such complementary payments are in line with subsection 15.b of the European Pillar of Social Rights (European Parliament, the Council of the European Union and the European Commission, 2017), which establishes that “[e]veryone in old age has the right to resources that ensure living in dignity”.
3. Effective prevention of tax evasion and avoidance, especially by large taxpayers, covering aggressive fiscal planning by multinationals and a review of the privileged treatment given to major corporate groups and capital income. In short, the idea is to prevent intra-Community fiscal competition from draining off tax revenue from MS.
4. Throughout the EU, or through the reinforced cooperation procedure if any country is irrevocably opposed, the social dimension of European integration should be strengthened by gradually harmonising social protection policies across MS, establishing minimum standards of cover for risk and financial effort. As indicated by the European Commission (2017), this would be a way of preserving the strength and stability of the Euro and preventing brusque changes in the living standards of citizens. Such common standards could be applied to labour markets, social provisions and certain areas of tax policy (e.g. corporation tax).
5. Consider directly communitarising provision by EU institutions some social protection functions, such as unemployment subsidies, healthcare or the fight against poverty and social exclusion. In this regard there is an interesting report by the IMF (Allard et al., 2013) on the potential for moving forward towards minimum standards of protection against unemployment across the Eurozone.
6. Conduct a painstaking assessment of the social policies of the MS to gauge their effectiveness in covering risks and needs and their effectiveness as mechanisms for redistributing income and wealth. The Social Protection Committee of the EU began to discuss and analyse the social policies of MS in 2015 through its annual reports, and could follow this up in greater depth.
7. Consider closing the Community market to products from countries that engage in social dumping or fail to provide sufficient protection for their workers, or setting an additional tax to cater for this. In a Communication dated 2001 (European Commission, 2001) the European Commission proposed “core labour standards” that could be developed through a Generalised Scheme of Preferences (GSP), given that the international standards and structures in place in economic and social fields suffer from imbalances at global scale (“market governance has developed more quickly than social governance”).
  In this regard, 2001 saw the approval of a Regulation of the Council (Council of the European Union, 2001) on the application of the GSP for 2002–2004 which stipulated a special framework of incentives to protect labour rights. The current wording (European Parliament and the Council of the EU, 2017) speaks of a “special incentive arrangement for sustainable development and good governance” and calls on potential beneficiary countries to sign up to the main UN and ILO conventions on human and labour rights, and to conventions on the environment and on the principles of governance. However, the standards cited are so basic that they do not solve the problem, and countries that trade with the EU would have to observe additional requirements to prevent social dumping. Again, there is a conflict of interests between MS: greater protection is highly important to those on the periphery (who specialise in conventional production activities in which they compete with developing countries), but the core countries, with complementary production structures, can benefit from the entry of highly-standardised, low-cost goods without such products affecting them in terms of competition.
8. Innovate by designing protection systems, employment policies and taxation frameworks that incentivise work and investment and at the same time prevent poverty from becoming chronic. At a time when rates of in-work poverty are high (EUROSTAT (2020) puts the figure at around 9% for the EU as a whole in 2019, with the highest rates being 15.4% in Rumania and 12.8% in Spain) because of involuntary part-time work, short-term contracts and low wages, innovative solutions need to be found for low-wage workers (e.g. supplementing their wages up to a level that enables them to make ends meet or making workers more productive so that they can earn more). The ultimate goal is to foster effort and proactive behaviour among citizens and prevent them from becoming demotivated or resigned to a life on benefits on the one hand, and to assure minimum welfare standards for the whole population on the other.

## Footnotes

1 Spanish President Rodríguez Zapatero answered that “the government has the intention to approve at the above-mentioned extraordinary Council of Ministers meetings, various regulatory changes to labour market legislation improving contracting flexibility to accelerate job creation” and reaffirmed “the Spanish government’s commitment to fiscal consolidation and structural reforms to foster growth in Spain”. For his part, the Italian government led by Monti quickly adopted the ECB letter – and the structural reforms it prescribed – as its roadmap. The government identified pension and labour policy as the stage where to show commitment to reform and acquire reputation by successfully tackling issues that had daunted the previous governments (Sacchia, 2015, p. 85).
